# Relationship between drug holiday of the antiresorptive agents and surgical outcome of medication-related osteonecrosis of the jaw in osteoporosis patients

**DOI:** 10.1038/s41598-022-15720-7

**Published:** 2022-07-07

**Authors:** Kota Morishita, Sakiko Soutome, Mitsunobu Otsuru, Saki Hayashida, Maho Murata, Miho Sasaki, Yukinori Takagi, Misa Sumi, Masahiro Umeda

**Affiliations:** 1 Department of Clinical Oral Oncology, Nagasaki University Graduate School of Biomedical Sciences, Nagasaki, Japan; 2 Department of Oral Health, Nagasaki University Graduate School of Biomedical Sciences, 1-7-1 Sakamoto, Nagasaki, 852-8588 Japan; 3 Department of Radiology and Biomedical Informatics, Nagasaki University Graduate School of Biomedical Sciences, Nagasaki, Japan

**Keywords:** Dental diseases, Oral medicine

## Abstract

A drug holiday of 3 months does not promote separation of sequestra and is not correlated with treatment outcomes after surgical therapy in osteoporosis patients who receive antiresorptive agents and who have medication-related osteonecrosis of the jaw. Medication-related osteonecrosis of the jaw (MRONJ) is a serious adverse effect of antiresorptive agents alone or in combination with immune modulators or antiangiogenic medications, in the absence of radiation exposure to the head and neck region. The effectiveness of surgical treatment for MRONJ has been reported, but the timing of the operation remains controversial. The purpose of this study was to clarify whether preoperative drug holidays of antiresorptive agents promote sequestrum separation and improve treatment outcomes in patients who receive low doses of antiresorptive agents. This retrospective study included 173 patients who received low-dose antiresorptive agents and underwent surgical therapy. The effects of a drug holiday on the separation of sequestra and treatment outcomes were analyzed using logistic and Cox regression analyses. Multivariate analysis revealed that administration of an antiresorptive agent for more than 4 years, a high number of lymphocytes, and an extensive osteolytic area were significantly correlated with separation of sequestra, but drug holiday did not promote sequestrum separation. Furthermore, a drug holiday of 90, 120 or 180 days did not show any improvement in treatment outcomes. The drug holiday of the antiresorptive agents for the treatment of MRONJ is unnecessary, and surgical therapy should be performed early.

## Introduction

Medication-related osteonecrosis of the jaw (MRONJ) is a serious adverse effect of antiresorptive agents, such as bisphosphonate (BP) and denosumab (Dmab). As a strategy for treating MRONJ, the position paper of the American Association of Oral and Maxillofacial Surgeons^1^ recommends the use of oral antimicrobial rinses in combination with antibiotic therapy for stages 0–1 and most stage 2 MRONJ^1^. The position paper of the Allied Task Force Committee of the Japanese Society for Bone and Mineral Research on BP-related osteonecrosis of the jaw also recommends conservative therapy for stages 0–1 and most stage 2 MRONJ and plural, long-term, and consecutive intravenous antimicrobial therapy for intractable cases^2^. In contrast, some studies have demonstrated the effectiveness of surgical therapy^3–17^. We also reported previously that the treatment outcome of surgical therapy was superior to that of conservative therapy in a propensity score matching analysis^18^.

Surgical therapy is enforced as a first-line treatment for MRONJ^19^ in our institutions, but there are differing opinions about the timing of the surgery. Kim et al. reported that a drug holiday before surgery was significantly correlated with good outcomes in 21 patients with MRONJ treated surgically and recommended that a drug holiday be initiated more than 4 months preoperatively^20^. In addition, Kaibuchi et al. reported that the cure rate of patients treated with surgery after separation of the sequestrum was higher than that of patients who underwent surgery without sequestrum separation^21^. Otto et al. stated that surgery is beneficial for mucosal healing; however, the optimal treatment sequence remains unclear^22^.

Consequently, many researchers believe that surgical therapy is more effective than conservative therapy in obtaining complete healing of MRONJ. However, it is controversial whether surgery should be performed early or after conservative treatment and a specific duration of the drug holiday. The purpose of this retrospective study was to clarify whether preoperative drug holidays of antiresorptive agents promote sequestra separation and improve treatment outcomes in patients receiving low-dose antiresorptive agents.

## Results

### Patient characteristics

Demographic factors, laboratory data, and imaging findings of the patients are summarized in Table 1. Twenty patients were male, and 153 were female, with a mean age of 78.7 years. The causative disease of BP administration was primary osteoporosis in 125 patients and secondary osteoporosis due to other diseases, such as rheumatoid arthritis, in 48 patients. Of these 173 patients, 36 received drug holidays of more than 90 days. Separation of sequestra was detected in 92 patients.

**Table 1:** Patient characteristics.

Variable		Number of patients/mean ± SD
Sex	Man	20
	Woman	153
Age	(years)	78.7 ± 9.31
Primary disease	Primary osteoporosis	125
	Others	48
MRONJ site	Upper jaw	50
	Lower jaw	123
MRONJ stage	Stage 1	13
	Stage 2	106
	Stage 3	54
Antiresorptive agent	BP	139
	Dmab	19
	BP → Dmab	15
Use of steroid	(−)	126
	(+)	47
Use of methotrexate	(−)	163
	(+)	10
Diabetes	(−)	150
	(+)	23
Duration of administration	< 4 years	67
	≧ 4 years	106
Duration of drug holiday before surgery	(days)	69.1 ± 168
Drug holiday ≧ 90 days	(−)	128
	(+)	36
	Unknown	9
Leukocytes	(/µL)	6510 ± 2021
Lymphocytes	(/µL)	1579 ± 729
Albumin	(g/dL)	3.78 ± 0.525
Creatinine	(mg/dL)	0.897 ± 0.814
Extent of osteolytic area	Localized	108
	Extensive	65
Periosteal reaction	(−)	133
	(+)	40
Separation of sequestrum	(−)	81
	(+)	92
Total		173

BP: bisphosphonate, Dmab: denosumab.

### Factors affecting the separation of sequestra

Of 92 patients in whom separation of sequestra was observed, 27 cases had a drug holiday of 90 days or more, 61 cases did not, and 4 cases had an unknown drug holiday. Univariate analysis revealed that seven variables, including primary osteoporosis, BP, no use of methotrexate, administration of antiresorptive agent for more than 4 years, the drug holiday of the antiresorptive agents for more than 90 days, diabetes, a high number of lymphocytes, and extensive osteolytic area, were significantly correlated with the separation of sequestra (Table 2). Multivariate logistic regression analysis with these seven factors revealed that administration of antiresorptive agent for more than 4 years (*p* = 0.006, odds ratio = 3.105, 95% confidence interval [95% CI] = 1.390–6.937), a high number of lymphocytes (*p* = 0.012, odds ratio = 1.001, 95% CI = 1.000–1.001), and extensive osteolytic area (*p* = 0.002, odds ratio = 3.710, 95% CI = 1.636–8.412) were significantly correlated with separation of sequestra (Table 3).

**Table 2:** Relationship between each variable and sequestrum separation (univariate analysis).

Variable		Sequestrum separation (−)	Sequestrum separation (+)	*p* value
Sex	Man	9	11	1.000
	Woman	72	81	
Age	(years)	77.4 ± 9.50	79.8 ± 9.01	0.101
Primary disease	Primary osteoporosis	51	74	0.011*
	Others	30	18	
MRONJ site	Upper jaw	24	26	0.868
	Lower jaw	57	66	
MRONJ stage	Stage 1	8	5	0.091
	Stage 2	54	52	
	Stage 3	19	35	
Antiresorptive agent	BP	59	80	0.011*
	Dmab	15	4	
	BP → Dmab	7	8	
Use of steroid	(−)	54	72	0.123
	(+)	27	20	
Use of methotrexate	(−)	73	90	0.047*
	(+)	8	2	
Diabetes	(−)	68	82	0.373
	(+)	13	10	
Duration of administration	< 4 years	38	29	0.043*
	≧ 4 years	43	63	
Drug holiday ≧ 90 days	(−)	67	61	0.004*
	(+)	9	27	
Leukocytes	(/µL)	6199 ± 1943	6773 ± 2068	0.077
Lymphocytes	(/µL)	1367 ± 691	1765 ± 713	0.001*
Albumin	(g/dL)	3.74 ± 0.565	3.82 ± 0.490	0.359
Creatinine	(mg/dL)	0.826 ± 0.314	0.956 ± 1.06	0.319
Extent of osteolytic area	Localized	60	48	0.004*
	Extensive	21	44	
Periosteal reaction	(−)	67	66	0.105
	(+)	14	26	
Total		81	92	

*Significant.

BP: bisphosphonate, Dmab: denosumab.

**Table 3:** Relationship between each variable and sequestrum separation (multivariate logistic regression).

Variable		*p* value	OR	95% CI
Primary disease	Others/primary osteoporosis	0.375	0.669	0.275–1.628
Antiresorptive agent	BP → Dmab/Dmab/BP	0.141	0.621	0.329–1.171
Use of methotrexate	(+)/(−)	0.324	0.361	0.048–2.733
Duration of administration	≧ 4 years/< 4 years	0.006*	3.105	1.390–6.937
Drug holiday ≧ 90 days	(+)/(− )	0.065	2.608	0.942–7.218
Lymphocytes	(/µL)	0.012*	1.001	1.000–1.001
Extent of osteolytic area	Extensive/localized	0.002*	3.710	1.636–8.412

*Significant.

BP: bisphosphonate, Dmab: denosumab, OR: odds ratio, 95% CI: 95% confidence interval.

### Factors affecting treatment outcomes

**Figure 1:**
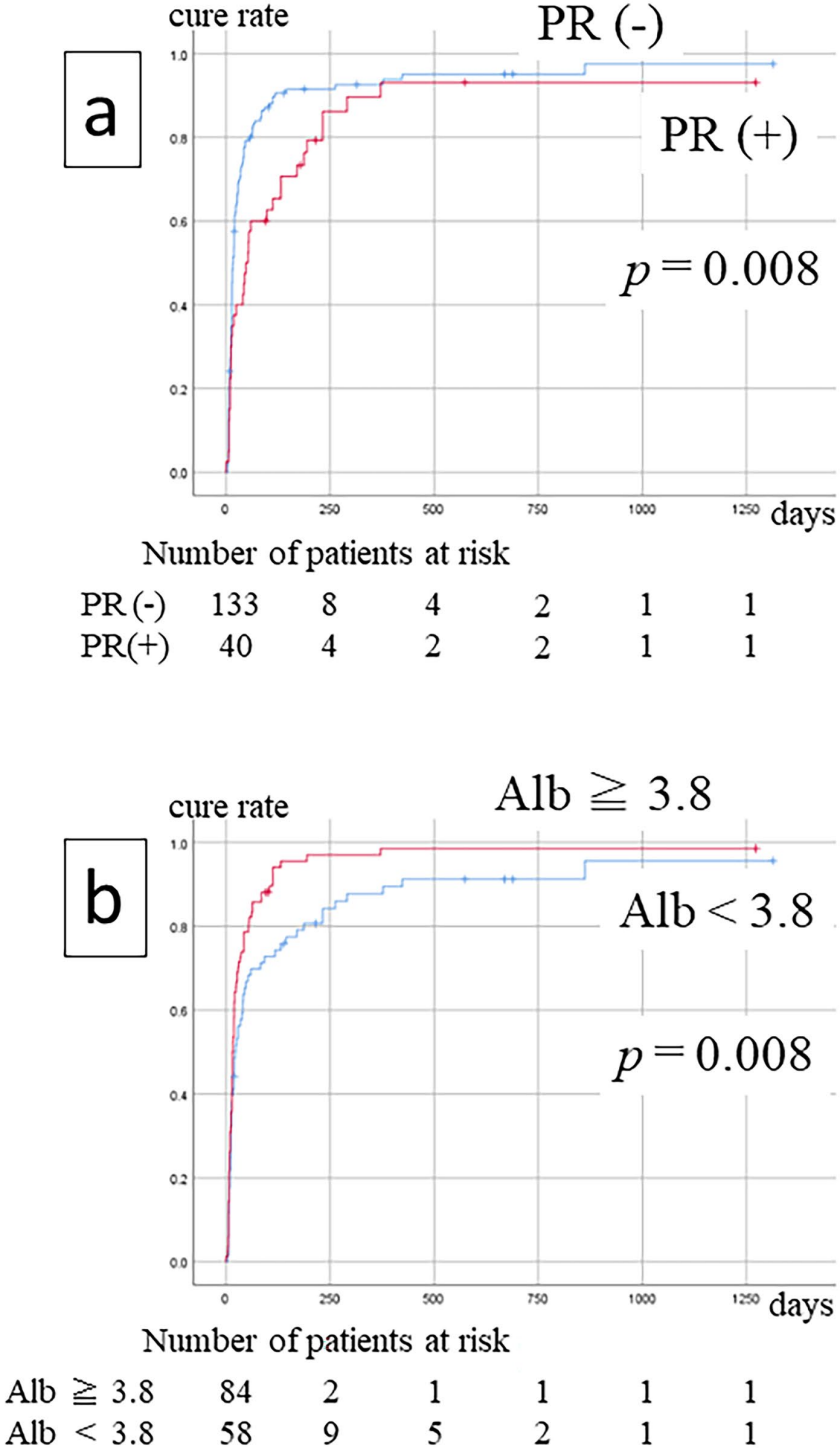
Factors related to the treatment outcomes of patients with medication-related osteonecrosis of the jaw. Patients with PR(+) showed significantly worse treatment outcomes than those with PR(−) (**a**). Patients with MRONJ cure rates in the Alb level (**b**). PR: periosteal reaction; Alb: serum albumin.

Univariate Cox regression analysis revealed that low serum albumin and periosteal reaction were significantly correlated with poor treatment outcomes. Multivariate Cox regression analysis of these two variables and drug holiday showed that serum albumin (*p* = 0.040, hazard ratio = 1.412, 95% CI = 1.016–1.963) and periosteal reaction (*p* = 0.032, hazard ratio = 0.649, 95% CI = 0.437–0.963) were significantly correlated with treatment outcome (Table 4). However, the drug holiday from the antiresorptive agent did not influence the treatment outcome. The Kaplan–Meier curve also showed a relationship between these two variables and treatment outcomes (Fig. 1).

**Table 4:** Relationship between each variable and treatment outcome (univariate and multivariate cox regression).

Variable		Univariate analysis			Multivariate analysis		
		*p* value	HR	95% CI	*p* value	HR	95% CI
Sex	Woman/man	0.956	0.987	0.610–1.597			
Age	(Years)	0.618	0.996	0.981–1.011			
Primary disease	Others/primary osteoporosis	0.660	0.924	0.651–1.312			
MRONJ site	Lower jaw/upper jaw	0.407	0.864	0.611–1.221			
MRONJ stage	Stage 3/2/1	0.300	0.869	0.667–1.133			
Antiresorptive agent	BP → Dmab/Dmab/BP	0.345	1.136	0.872–1.479			
Use of steroid	(+)/(−)	0.439	0.869	0.610–1.239			
Use of methotrexate	(+)/(−)	0.440	1.305	0.664–2.566			
Diabetes	(+)/(−)	0.458	0.840	0.530–1.332			
Duration of administration	≧ 4 years/< 4 years	0.763	1.050	0.763–1.446			
Drug holiday ≧ 90 days	(+)/(−)	0.480	1.145	0.787–1.666	0.957	1.011	0.679–1.506
Leukocytes	(/µL)	0.390	1.004	0.995–1.012			
Lymphocytes	(/µL)	0.075	1.021	0.998–1.044			
Albumin	(g/dL)	0.020*	1.472	1.064–2.036	0.040*	1.412	1.016–1.963
Creatinine	(mg/dL)	0.392	0.910	0.734–1.129			
Extent of osteolytic area	Extensive/localized	0.115	0.769	0.555–1.066			
Periosteal reaction	(+)/(−)	0.009*	0.603	0.412–0.883	0.032*	0.649	0.437–0.963
Separation of sequestrum	(+)/(−)	0.106	1.298	0.946–1.781			

*Significant.

BP: bisphosphonate, Dmab: denosumab, HR: hazard ratio, 95% CI: 95% confidence interval.

**Figure 2:**
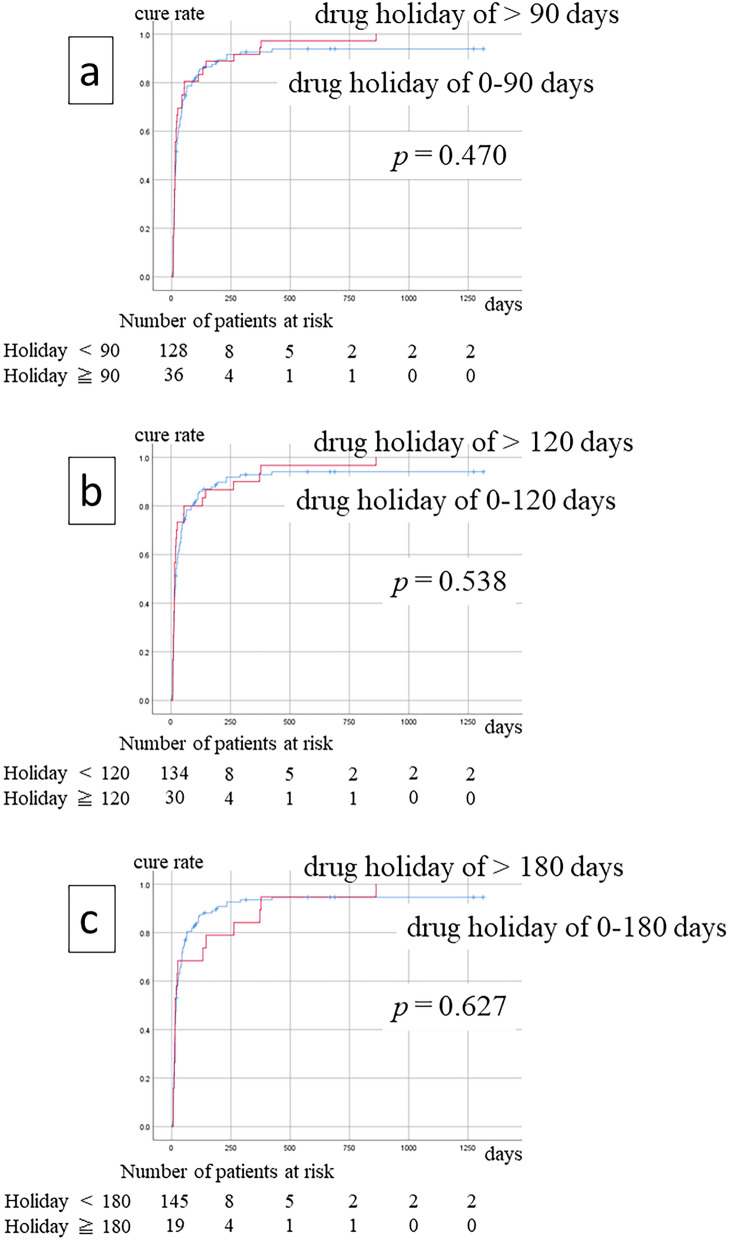
Treatment outcomes did not improve after 90 (**a**), 120 (**b**), or 180 (**c**) days of drug holiday.

Figure 2 shows the effects of drug holidays on treatment outcomes. Drug holidays of 90, 120, or 180 days did not show any improvement in treatment outcomes.

## Discussion

The results of this study suggested that the drug holiday of the antiresorptive agents in MRONJ patients who plan to undergo surgery showed no significant effects on improving treatment outcomes.

Treatment strategies for MRONJ remain controversial. First-line treatment of MRONJ is conservative therapy for stages I and II with some exceptions according to the Position Paper of AAOMS and JSOMS^1,2^. These position papers recommend drug holidays before invasive dental treatments, such as tooth extraction. However, they also stated that there were no studies to support these recommendations but their approach was based on bone physiology and pharmacokinetics of the antiresorptive agents, and that prospective studies were required to establish the efficacy of drug holidays in decreasing the risk of MRONJ. Although conservative therapy is beneficial because it avoids surgical aggression, in many cases, it leads to poor outcomes. In addition, conservative therapy is administered over a long period of time and may decrease the quality of life of patients. Therefore, there is a consensus that surgical treatment should be performed to completely cure the symptoms, including bone exposure^3–17^.

However, there are various opinions regarding when to perform MRONJ surgery. Kim et al. reported that 12 out of 21 surgically managed patients showed a favorable prognosis and nine patients relapsed, and that drug holiday was the only prognostic factor in the surgical management group. They also stated that because of the poor prognosis in patients who had drug holidays of 1.5–4 months prior to surgical treatment, a drug holiday of more than 4 months preoperatively should be recommended^20^. Kaibuchi et al. reported that the cure rate of sequestrectomy after separation of sequestrum while providing conservative therapy is higher than that in cases where sequestrum separation is not detected^21^. However, they did not mention whether sequestrum separation was promoted by the drug holiday of the antiresorptive agents. Otto et al. described in a recent review that conservative treatment improves symptoms in patients with MRONJ but is difficult to cure by covering the mucous membrane, and surgery may be beneficial when symptom management and mucosal healing are the ultimate goals. However, they also stated that the optimal treatment sequence remains unclear^22^. Thus, no study has examined the association between the drug holiday of the antiresorptive agents and sequestrum separation or treatment outcomes in detail.

Osteomyelitis of the jaw caused by bacterial infection can be divided into early, advanced, sequestrum-formation, and sequestrum-separation phases^23^. As the disease becomes chronic over a long period of time, sequestrum forms, and the immune system begins to eliminate it from the bone tissue as a foreign substance. In the current study, the frequency of sequestrum separation in patients who discontinued antiresorptive agents was high in the univariate analysis, but the difference was not significant in the multivariate analysis. This suggests that the drug holiday of the antiresorptive agents did not promote separation of the sequestra, but rather that the frequency of separation of sequestra was high in patients who were in good general health with good bone condition and who could then safely take a drug holiday. In fact, the factors that were significantly associated with the separation of sequestra in the multivariate analysis were long-term administration of antiresorptive agent, extensive osteolytic area, and high number of lymphocytes. In cases in which antiresorptive agents had been administered for a long period of time, it is possible that the patient had asymptomatic chronic osteomyelitis and a long period of illness before the diagnosis of MRONJ. Then, the sequestrum may have already begun to separate by the time the diagnosis was made. Patients with a high number of lymphocytes may be more prone to sequestrum separation, as the immune system can better eliminate foreign substances. In the case of extensive osteolysis, chronic inflammation may have been present for a long period of time before bone resorption extends, and the mechanism for removing foreign substances may have been working to separate the sequestrum for a long time. In other words, it is thought that patients who develop MRONJ for a long period of time and who show disease progression, but whose systemic immunity has been maintained, have a tendency to separate sequestra as a protective reaction of the body. In contrast, patients who develop MRONJ for a short period of time and who have small lesions or who develop MRONJ for a longer period of time but are immunocompromised will not show sequestra separation. Because patients who have drug holidays are often those with stable osteoporosis following long-term treatment with antiresorptive agents and those who are immunocompetent, the frequency of sequestrum separation may have been high in patients who discontinued antiresorptive agents in the univariate analysis.

BPs have a high affinity for hydroxyapatite, and when they enter the bloodstream, most are deposited in the bone, where the BPs are taken up by osteoclasts during bone resorption. BP that has been deposited in the bone has a remarkably long half-life; the half-life of alendronic acid is estimated to be more than 10 years^24^. Dmab is a human monoclonal immunogloblin 2 antibody that acts against the RANKL protein. RANKL plays an important role in osteoclast differentiation and maturation, and when Dmab inhibits the binding of RANKL to its receptor, RANK, osteoclast activation and differentiation into mature osteoclasts is suppressed^25^. Dmab is not deposited in the bone and has a half-life of about 1 month in blood; it is thought to have a shorter inhibitory effect on osteoclasts than BP, but Dmab is more potent than BPs in inhibiting osteoclasts. In fact, a recent systematic review showed that the incidence of MRONJ is higher with Dmab than that with BPs^26^. In any case, it is questionable whether osteoclast activity can be restored and bone metabolism normalized after a few months of drug holiday. Although the American Association of Oral and Maxillofacial Surgeons^1^ and Japanese position paper^2^ recommend that BPs should be discontinued for 2 months prior to invasive dental procedures, such as tooth extraction in osteoporotic patients receiving BPs, our study of 2458 extractions in osteoporotic patients receiving oral BPs reported that a drug holiday of 3 months prior to extraction did not reduce the incidence of MRONJ^27^. Based on these findings, it is unclear whether separation of sequestra will be promoted even after several months’ the drug holiday of the antiresorptive agents.

We performed a multivariate analysis of factors influencing treatment outcomes. As in previous reports^28^, the presence of a periosteal reaction significantly decreased the treatment outcomes, and a low serum albumin level was also a poor prognostic factor. There was no association between drug holidays and treatment outcomes, and drug holidays of 3, 4 or 6 months had no effect on postoperative outcomes.

This study had some limitations. First, since it was a retrospective study and a multivariate analysis was carried out, there may have been unknown confounding factors. Second, it was a cross-sectional study and did not chronologically observe, through imaging, sequestrum separation. Therefore, the accuracy of the results was not clear. However, this study is the first to examine the relationship between drug holiday of the antiresorptive agents and sequestrum separation, and few studies have investigated the relationship between the drug holiday and treatment outcomes. We believe that prospective observational studies are necessary to draw clearer conclusions.

## Conclusion

In patients with MRONJ who receive antiresorptive agents, a drug holiday of 3 months did not promote separation of sequestra and was not correlated with treatment outcome after surgical therapy. These findings suggest that the drug holiday of the antiresorptive agents for the treatment of MRONJ is unnecessary, and surgical therapy should be performed early.

## Methods

### Patients

In total, 196 patients with MRONJ caused by low doses of antiresorptive agents were treated at Nagasaki University between 2011 and 2019. Twenty-three patients who were treated only with conservative therapy were excluded from the study, and 173 patients who were treated with surgical therapy were enrolled in this retrospective study. As a method of surgery, extensive surgery, which removes not only necrotic bone but also surrounding healthy bone was performed^18^.

### Variables

Various clinical factors were examined retrospectively based on the patients’ medical records. These factors included age; sex; site of MRONJ (upper or lower jaw); stage of MRONJ (AAOMS^1^); primary disease (primary osteoporosis or others, such as rheumatoid arthritis); use of methotrexate; type of antiresorptive agent used (BP or Dmab); duration of antiresorptive agent administration; the drug holiday of the antiresorptive agents; separated sequestrum visible on computed tomography just before surgery; extent of osteolytic area; periosteal reaction; use of steroids; diabetes; levels of leukocytes, lymphocytes, serum albumin, and creatinine; and treatment outcomes. Regarding the extent of the osteolytic area, bone resorption that reached the mandibular canal or maxillary sinus was considered to be ‘extensive’. If computed tomography showed that the necrotic bone had completely, or almost, separated from the jawbone, the sequestrum was determined to be separate. For treatment outcomes, ‘cure’ was determined to have been achieved when all symptoms and signs, including the exposed bone, had disappeared. In cases judged to be cured, the time to cure was recorded and used for Cox regression analysis. If all the symptoms disappeared but then relapsed, it was considered “non-healing”. The observation discontinuation period is shown in Figs. 1 and 2.

### Statistical analysis

All statistical analyses were performed using the SPSS software (version 26.0; Japan IBM Co., Ltd., Tokyo, Japan). The correlation between each variable and sequestrum separation was analyzed using Fisher’s exact test and one-way analysis of variance, followed by logistic regression with factors that were significant in the univariate analysis. Next, the cumulative cure rate of MRONJ was calculated using the Kaplan–Meier method and was analyzed using univariate and multivariate Cox regression analyses. All analyses were two-tailed, and *p-*values < 0.05 were considered statistically significant.

### Ethical approval

The study protocol conformed to the ethical guidelines of the Declaration of Helsinki and the Ethical Guidelines for Medical and Health Research involving Human Subjects by the Ministry of Health, Labor, and Welfare of Japan. Ethical approval was obtained from the Institutional Review Board (IRB) of Nagasaki University Hospital (No. 21021509). Japanese law does not require individual informed consent from participants in non-invasive observational trials such as the present study. Therefore, the need for informed consent was waived according to the instruction of IRB of Nagasaki University Hospital. As this was a retrospective study, patient identifiable information was removed, and the research plan was published on the homepages of the participating hospitals websites, along with an opt-out option in accordance with the IRB of Nagasaki University Hospital instructions.

### Consent for publication

Patient-identifiable information was removed, and the research plan was published on the homepages of the participating hospitals’ websites, together with an opt-out option in accordance with the Institutional Review Board’s instructions.

## Data Availability

The data that support the findings of this study are available from the corresponding author upon reasonable request.
